# Chromium picolinate and chromium histidinate protects against renal dysfunction by modulation of NF-κB pathway in high-fat diet fed and Streptozotocin-induced diabetic rats

**DOI:** 10.1186/1743-7075-9-30

**Published:** 2012-04-08

**Authors:** Mustafa Yavuz Selcuk, Bilge Aygen, Ayhan Dogukan, Zeynep Tuzcu, Fatih Akdemir, James R Komorowski, Mustafa Atalay, Kazim Sahin

**Affiliations:** 1 Department of Nephrology, School of Medicine, Firat University, Elazig, Turkey; 2 Department of Biology, Faculty of Science Firat University, Elazig, Turkey; 3 Department of Animal Nutrition, Faculty of Veterinary Science, Firat University, Elazig 23119, Turkey; 4 Technical Services and Scientific Affairs, Nutrition 21 Inc., 4 Manhattanville Road, Purchase, NY, USA; 5 Institute of Biomedicine, Physiology, University of Eastern Finland, Kuopio, Finland; 6 Department of Nutrition, Faculty of Fisheries, Inonu University, Malatya, Turkey

**Keywords:** Chromium picolinate/histidinate, NF-κB, IκB, Nrf2, HNE, Kidney

## Abstract

**Background:**

Diabetic nephropathy is one of major complications of diabetes mellitus. Although chromium is an essential element for carbohydrate and lipid metabolism, its effects on diabetic nephropathy are not well understood. The present study was conducted to investigate the effects of chromium picolinate (CrPic) and chromium histidinate (CrHis) on nuclear factor-kappa B (NF-κB) and nuclear factor-E2-related factor-2 (Nrf2) pathway in the rat kidney.

**Methods:**

Male Wistar rats were divided into six groups. Group I received a standard diet (8% fat) and served as a control; Group II was fed with a standard diet and received CrPic; Group III was fed with a standard diet and received CrHis; Group IV received a high fat diet (HFD, 40% fat) for 2 weeks and then were injected with streptozotocin (STZ) (HFD/STZ); Group V was treated as group IV (HFD/STZ) but supplemented with CrPic for 12 weeks. Group VI was treated as group IV (HFD/STZ) but supplemented with CrHis.

**Results:**

The increased NF-κβ p65 in the HFD/STZ group was inhibited by CrPic and CrHis supplementation (*P *< 0.05). In STZ-treated rats, a significant decrease in levels of nuclear factor of kappa light polypeptide gene enhancer in B-cells inhibitor, alpha (IκBα) was found in kidney tissues when compared to control rats (*P *< 0.05). A significant increase in the levels of IκBα was observed in CrPic- and CrHis-treated rats when compared with STZ-treated rats. Renal Nrf2 levels were significantly decreased in diabetic rats compared with the control rats. There was a higher tendency for increase of kidney Nrf2 level and decrease in kidney NFκBp65 levels and 4- hydroxyl nonenal (4-HNE) protein adducts (*P *< 0.05) in diabetic rats.

**Conclusion:**

Our result show that in kidney tissue CrHis/CrPic increases Nrf2 level, parallelly decreases NF-κB and partially restores IκBα levels in HFD/STZ group, suggesting that CrPic and CrHis may play a role in antioxidant defense system via the Nrf2 pathway by reducing inflammation through NF-κβ p65 inhibition. Moreover, a greater reduction in NF-κB expression and greater increases in expressions of IκBα and Nrf2 in diabetic rats supplemented with CrHis than rats supplemented with CrPic suggest that CrHis has more favorable effects than CrPic.

## Background

Nutrition plays an important role in the development and also in the prevention of cancer, cardiovascular diseases, and diabetes. A high-fat diet can induce obesity and metabolic disorders, insulin resistance, dyslipidemia, and hypertension in rodents and in humans [1-3]. High-fat diets (HFD) may induce changes not only in energy metabolism but also in liver, kidney and brain function.

Diabetes mellitus, a well-known endocrine metabolic disorder, is disease characterized by high levels of blood glucose and multiple tissue complications, resulting in nephropathy, neuropathy and retinopathy [4]. Oxidative stress and disrupted redox regulation play an important role in the pathogenesis of diabetes and one of the major complications is renal and, in addition to cerebrovascular and ocular complications [5-8]. The production of tumor necrosis factor (TNF)-α and expression of nuclear transcription factor kappa B (NFκB) can be stimulated by over production of reactive oxygen species (ROS) [9]. Inflammatory proteins may also participate in the pathogenesis of insulin resistance and its complications [10] and therefore, mechanisms by which insulin resistance occurs are explained by excessive activities in the NFκB pathway and of inflammatory cytokines [11-13]. Activated the inhibitor of NF-κB kinase (IKK), caused by cytokines, hyperglycemia and elevated free fatty acids (FFAs), results in the nuclear factor of kappa light polypeptide gene enhancer in B-cells inhibitor, alpha (IκBα) polyubiquitination and proteosomal degradation, and subsequent release of NFκB, especially p50/p65, to the nucleus where it can bind to the response element of target genes involved in the inflammatory response [14-16]. In return sustained activation of NFκB caused by overexpression of IKK in the liver leads to insulin resistance [14].

Some strategies to alleviate insulin resistance by nutritional supplements appear to improve insulin sensitivity in many individuals and improve glycemic control in diabetics [17]. Chromium (Cr) is required for normal carbohydrate, protein and lipid metabolism and its deficiency has been implicated as one of the causes of diabetes mellitus [18-20]. Supplementations of available Cr chelates with picolinic acid (CrPic) [21,22] and histidinate (CrHis) [23] have been shown to exert beneficial effects for the management of type-2 diabetes, as reflected by a decline in insulin response.

Several studies reported that CrPic and CrHis may enhance insulin receptor binding [24], increase the number of insulin receptors [25] and insulin receptor phosphorylation [26], resulting in the reduction of insulin resistance in peripheral tissues [27]. One of the intracellular proteins influencing the insulin receptor is the oligopeptide low molecular weight chromium binding substance (LMWCr, apochromodulin) which is widely distributed in the liver, kidneys, spleen, intestine, testicles and brain [28,29]. This peptide, which activates tyrosine kinase, depends on Cr concentration and promotes insulin receptor activity.

Although numerous studies have been published examining the health aspects of chromium on humans and animals, there have been scarce studies to investigate the effects of CrHis on the IκB/NF-κB pathway or Nrf2 response in diabetic nephropathy. Therefore, the current study was performed to investigate the effect of CrHis/CrPic supplementation on changes in IκB/NF-κB pathway and Nrf2 levels in the diabetic nephropathy.

## Methods

### Animals and diets

Male Wistar rats (n = 90, 8 wk-old) weighing 200-250 gr were purchased from Firat University Laboratory Animal Research Center (Elazig, Turkey). These animals were reared at the temperature of 22 ± 2°C, humidity of 55 ± 5%, and with a 12/12 h light/dark cycle. The experiment was conducted under the protocol approved by the Ethical Committee of Firat University. All procedures involving rats were conducted in strict compliance with relevant laws, the Animal Welfare Act, Public Health Services Policy, and guidelines established by the Institutional Animal Care and Use Committee of the University. Rats consumed a standard diet and tap water ad libitum. Blood samples were collected from the tail vein of each rat for the measurement of biochemical efficacy markers. Rats were randomly assigned to treatment groups. The rats were fed with standard (control) diet (8% fat) or high fat diet (HFD) containing 40% fat for 12 weeks, and administered with either levels of CrPic and CrHis (Nutrition 21, Inc. NY, USA).

Ingredients and chemical composition of the basal (control) diet are shown in Table 1. The diets were stored at 4°C cold chamber. Animals were fed either with a normal diet consisting of 8% fat or a HFD consisting of 40% fat. CrPic and CrHis (Nutrition 21, Inc., Purchase, NY, USA) were dissolved in water and administered at a concentration of 22 μg CrPic and CrHis were delivered via drinking water for 12 weeks (providing 8 mcg Cr/day), which is an equivalent dose of 560 μg Cr for a 70 kg adult human.

**Table 1:** Composition of diets (g/kg diet) fed to rats

Ingredients	Regular Diet	High Fat Diet
Casein	200.0	200.0

Starch	615.0	145.0

Sucrose	-	150.0

Corn oil	80.0	-

Beef tallow	-	400.0

Cellulose	50.0	50.0

Vitamin-Mineral Premix*	50.0	50.0

DL-Methionine	3.0	3.0

Choline cloride	2.0	2.0

Chromium, mg/kg	0.066	0.097

*The vitamin-mineral premix provides the following (per kg): all-*trans*-retinyl acetate, 1.8 mg; cholecalciferol, 0.025 mg; all-*rac*-a-tocopherol acetate, 12,5 mg; menadione (menadione sodium bisulfate), 1.1 mg; riboflavin, 4.4 mg; thiamine (thiamine mononitrate), 1.1 mg; vitamin B-6, 2.2 mg; niacin, 35 mg; Ca-pantothenate, 10 mg; vitamin B-12, 0.02 mg; folic acid, 0.55 mg; *d*-biotin, 0.1 mg. manganese (from manganese oxide), 40 mg; iron (from iron sulfate), 12.5 mg; zinc (from zinc oxide), 25 mg; copper (from copper sulfate), 3.5 mg; iodine (from potassium iodide), 0.3 mg; selenium (from sodium selenite), 0.15 mg; choline chloride, 175 mg

### Experimental design and induction of type II diabetes

A rat model of type-2 diabetes which was created by feeding with a HFD and STZ treatment, developed by Reed et al. [30]. This model provides a novel animal model for type-2 diabetes that is applicable to the human syndrome making it suitable for testing antidiabetic compounds. In using such a model, the increased hyperglycemia after STZ injection in high-fat fed rats was not due to a greater decline in B-cell function [30,31]. The animals of the present work were divided into 6 groups as: Group I: rats were fed with a standard diet (8% fat); Group II: rats were fed with standard diet and received CrPic; Group III: rats were fed with standard diet (8% fat) and received CrHis; Group IV (HFD/STZ) (rats were fed with a HFD (40% fat) for 2 weeks and then injected with STZ (40 mg/kg i.p.); Group V (HFD/STZ + CrPic) (rats were fed with a high-fat diet (40% fat) for 2 weeks and then injected with STZ (40 mg/kg i.p.) and received CrPic; Group VI (HFD/STZ + CrHis) (rats were fed a high-fat diet (40% fat) for 2 weeks and then injected with STZ (40 mg/kg i.p.) and received CrHis. CrHis and CrPic were included into water and administered at a concentration of 22 μg CrPic and CrHis 12 weeks.

Before STZ injection, glucose concentrations of rats were measured and compared to controls. After STZ injection, animals exhibiting fasting glucose levels > 140 mg/dl were considered as STZ diabetic; resembling type-2 diabetes in human. CrPic and CrHis were then administered.

### Western blot analyses

In all groups, rats were sacrificed by cervical dislocations and kidneys were promptly removed. Protein extraction was performed as follows. The sample was homogenized in ice-cold in 1 ml of hypotonic buffer A [10 mM HEPES (pH 7.8), 10 mM KCl, 2 mM MgCl2, 1 mM DTT, 0.1 mM EDTA, 0.1 mM phenylmethylsulfonyl-fluoride (PMSF)]. A solution of 80 μl of 10% Nonidet P-40 (NP-40) was added to the homogenates, and the mixture was centrifuged for 2 min at 14,000 g. The supernatant was collected as a cytosolic fraction for the assays of IκBα and 4- hydroxyl nonenal (4-HNE) adducts. The precipitated nuclei were washed once with 500 μl of buffer A plus 40 μl of 10% NP-40, centrifuged, resuspended in 200 μl of buffer C [50 mM HEPES (pH 7.8), 50 mM KCl, 300 mM NaCl, 0.1 mM EDTA, 1 mM DTT, 0.1 mM PMSF, 20% glycerol] and centrifuged for 5 min at 14,800 g. The supernatant containing nuclear proteins was collected for Nrf2 and NFκB p65 [32]. Concentration of the protein was determined according to the procedure described by Lowry et al. [33] using a protein assay kit supplied by Sigma, St. Louis, MO, USA. Sodium dodecyl sulfate-polyacrylamide gel electrophoresis sample buffer containing 2% *β*-mercaptoethanol was added to the supernatant. Equal amounts of protein (50 μg) were electrophoresed and subsequently transferred to nitrocellulose membranes (Schleicher and Schuell Inc., Keene, NH, USA). Nitrocellulose blots were washed twice for 5 min each in PBS and blocked with 1% bovine serum albumin in PBS in room temperature for 1 h prior to application of the primary antibody. The antibody against Nrf2 and 4-HNE adducts were the purchased from Santa Cruz Biotechnology, Inc. (Santa Cruz, CA, USA) and from 4 Alpha Diagnostics (San Antonio, TX) respetively. Antibody against IκBa and NF-κB p65 were purchased from Abcam (Cambridge, UK). Primary antibody was diluted (1:1000) in the same buffer containing 0.05% Tween-20. The nitrocellulose membrane was incubated overnight at 4°C with protein antibody. The blots were washed and incubated with horseradish peroxidase-conjugated goat anti-mouse IgG (Abcam, Cambridge, UK). Specific binding was detected using diaminobenzidine and H_2_O_2 _as substrates. Protein loading was controlled using a monoclonal mouse antibody against β-actin antibody (A5316; Sigma). Blots were performed at least three times to confirm the data reproducibility. Bands were analyzed densitometrically using an image analysis system (Image J; National Institute of Health, Bethesda, USA).

### Statistical analysis

Sample size was calculated based on a power of 85% and a *P *value of 0.05. Given that assumption, a sample size of 10 rats per treatment was calculated. The data were analyzed using the General Linear Model (GLM) procedure of SAS software [34]. Least square treatments were compared if a significant F statistic (5% level of P) was detected by analysis of variance. Treatments were also compared using student's unpaired *t *test for comparison of individual treatment. *P *< 0.05 was considered to be statistically significant. Between group differences in latencies were analyzed by the analysis of variance for repeated measurements (ANOVAR) followed by Fisher's post hoc test for all groups.

## Results

**Figure 1:**
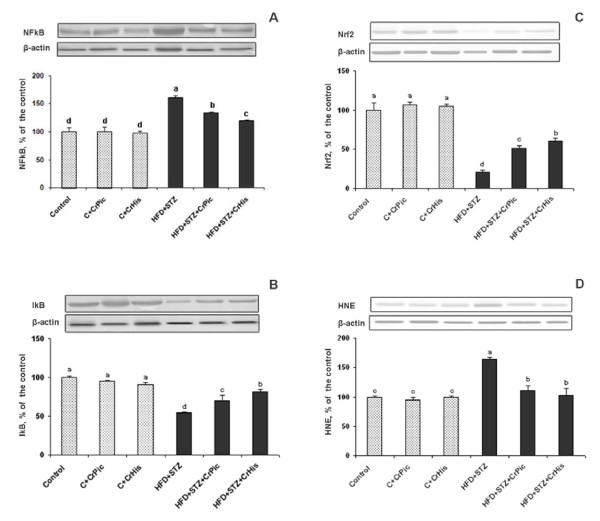
**Western blot analysis of NF-κBp65, IκB, Nrf2 and 4-HNE protein adducts in kidney homogenates of controls and chromium-treated rats**. Densitometric analyses of these bands are represented as percent of control. Values are means ± standard error of the mean. Blots were repeated at least 3 times (n = 3) and a representative blot is shown. Actin was included to ensure equal protein loading. Data points with different superscripts are significantly different at the level of P < 0.05 by Fisher's comparison test. Control: no treatment; CrPic: Fed with standard diet and received CrPic; C + CrHis: fed with standard diet (8% fat) and received CrHis; HFD + STZ: received a HFD (40% fat) for 2 weeks and then were injected with streptozotocin (STZ) on day 14 (STZ,40 mg/kg i.p); HFD + STZ + CrPic: Treated with HFD/STZ but supplemented with CrPic. HFD + STZ + CrHis: Treated with HFD/STZ but supplemented CrHis. The mean bars with different letters differ (P < 0.05).

Figure 1 shows the effects of CrPic and CrHis on NF-κβ, IκBα, Nrf2 levels and 4-HNE protein adducts of kidney in rats. In this study (Figure 1A), the kidney NF-κβ p65 subunit level increased significantly in STZ and HFD groups. After oral administration of CrPic and CrHis, kidney NF-κB level was not altered in rats fed a regular diet, but significantly decreased in diabetic rats. When Cr was supplemented as CrHis, compared to CrPic, there was a greater decrease (*P *< 0.05) in kidney NF-κB level in diabetic rats. Renal IκBα level assessed by Western blotting and were significantly decreased in diabetic rats compared with those in control rats (*P *< 0.05), where as CrPic- and CrHis treatment significantly offset these increases in diabetic rats (*P *< 0.01) (Figure 1B). In diabetic animals the level of IκBα was significantly increased by CrHis as compared to the similarly treated CrPic groups. The protein levels of Nrf2 in the renal tissues were also determined by Western blot analysis (Figure 1C). Based on the band densities, renal Nrf2 level was significantly decreased in diabetic rats compared with the control rats. However, these decreases were partially compensated by the supplements of CrPic and CrHis. Compared to CrPic, CrHis is more effective in increasing these proteins. When Cr was supplemented in CrHis form, compared to its CrPic form, there was a greater increase (*P *< 0.05) in kidney Nrf2 level in diabetic rats. However, level of these proteins in kidney was not affected by CrPic and CrHis supplementation in control rats fed a normal diet. The kidney 4-HNE protein adducts increased significantly in STZ and HFD groups. After oral administration of CrPic and CrHis, kidney 4-HNE protein adducts were not altered in rats fed regular diet, but were significantly decreased in diabetic rats (Figure 1D). Taken all together, CrPic and CrHis effects were similar when rats fed normal diet, but CrHis was superior to CrHis, as reflected by a greater reduction in NF-κB levels and 4-HNE protein adducts and greater increases in the levels of IκBα and Nrf2 in diabetic rats.

## Discussion

Chromium supplementation as chromium chloride (CrCl_3_) [35,36], chromium niacinate (Cr-N), CrPic [37] and CrHis [22] has been shown to inhibit the increase in inflammatory markers and oxidative stress levels in cultured monocytes exposed to high glucose levels. However, no research was reported in the literature for the effects of CrPic and CrHis supplementation on the kidney levels of NFκB, IκBα, Nrf2 and 4-HNE protein adducts in an animal model of diabetes. The results of the present work showed that CrPic and CrHis decreased NF-κB level in kidney of the rats with HFD/STZ-induced diabetes, indicating that CrPic and CrHis decreases 4-HNE protein adducts via the Nrf2/ARE-mediated pathway. In parallel with the results of the present work, Kuhad et al. [38] reported that NFκβ p65 subunit was significantly elevated in the kidneys of diabetic animals. These results are consistent with previous studies showing that CrPic supplementation improves renal function in STZ-diabetic rats [39,40]. Similar to the results of the present work, other reports have shown that diabetes causes alterations in the synthesis or concentrations of cytokines [36,37,41]. Chen et al. [42] showed that the levels of NF-κB p65 in kidneys were distinctly increased in lower dose STZ-induced diabetic rat model fed with a high-fat diet. In addition, Bi et al. [16] reported that insulin treatment inhibits liver NF-κB activity and inflammatory cytokine responses involved in the amelioration of insulin resistance in diabetic rats. Jain et al. [37] reported that Cr-N supplementation lowered the blood levels of tumor necrosis-α (TNF-α), Interleukin -6 (IL-6), c-reactive protein (CRP) and cholesterol, In addition CrPic supplementation caused a decrease in TNF-α, IL-6 and lipid peroxidation in rats [37].

Nrf2 controls the antioxidant response element (ARE)-dependent gene regulation in response to oxidative stress and contributes to protection against to the disorders related to oxidative stress [43,44]. In the present work, Nrf2 levels in the HFD/STZ-induced kidney injury group was lower than those of controls, whereas CrPic and CrHis treatment induced level of Nrf2 and enhanced nuclear translocation and subsequent ARE binding (Figure 1C). This finding shows that CrHis and CrPic may be involved in stabilization and maintaining levels of Nrf2. Palsamy and Subramanian [45] reported that during hyperglycemia-mediated oxidative stress, the expression of Nrf2 and its downregulatory enzymes like heme oxygenase- 1 were significantly decreased in the renal tissues of diabetic rats. CrHis was shown to reverse the deficits associated with obesity via up regulating the expression of Nrf2 and HO-1 in the rats fed HFD [46]. However, there are no previous studies investigating the effects of Cr supplementation on the Nrf2 in diabetic nephropathy comparable with this study.

Oxidative stress associated with overproduction of ROS plays an important role in the development of diabetic complications including diabetic nephropathy [47]. These modifications can cause some morphological and functional disorders in the kidney of diabetic patients. The reaction of ROS with membrane lipids causes the formation of lipid peroxidation products including several aldehydic compounds, one of which is highly toxic and called as 4- HNE adducts. 4-HNE adducts, an indicator of lipid peroxidation, in kidney of diabetic rats were decreased when dietary CrHis or CrPic was supplemented. CrHis supplementation did not alter these parameters in non-diabetic rats. The current study appears to be the first to examine the specific association between dietary CrPic/CrHis intake and 4-HNE in diabetic rats. Significantly lower levels of 4- HNE adduct were observed in diabetic animals receiving Cr supplementation. These findings indicate significant positive associations between Cr intake and 4- HNE adducts for diabetic rats. These results confirm previous findings that the level of HNE is altered in diabetes, resulting in increased susceptibility of the tissues to injury [48]. CrHis [22,46] and CrPic [37], playing an important role against insulin resistance, is postulated to augment antioxidant defense system. Previous studies have also demonstrated that CrCl_3 _supplementation inhibited the increase in TNF-α and oxidative stress levels in cultured monocytes exposed to high glucose levels [35,36]. Similarly, Jain et al. [37] reported that CrPic supplementation showed a decrease in lipid peroxidation, TNF-α, IL-6 in rats. Preuss et al. [39] also reported a decrease in hepatic thiobarbituric acid-reactive substances (TBARS) formation by supplementation of CrPic and Cr nicotinate in rats.

## Conclusion

The results of this study provide further evidence that CrPic and CrHis may have a protective role against diabetic nephropathy through the Nrf2 pathway and also through an anti-inflammation effect by NF-κB inhibition. Moreover, a greater reduction in NF-κB level and greater increases in the levels of IκBα and Nrf2 in diabetic rats supplemented with CrHis than rats supplemented with CrPic suggest that CrHis has more favorable effects than CrPic.

## Abbreviations

ARE: Antioxidant response element; CrHis: Chromium histidinate; CRP: C-reactive protein; CrPic: Chromium picolinate; FFA: Free fatty Acid; HFD: High fat diet; HNE: 4-hydroxynonenal adducts; HO-1: Hemeoxygenase-1; IL-6: Interleukin 6; IκB: Nuclear factor of kappa light polypeptide gene enhancer in B-cells inhibitor alpha; IKK: Inhibitor of NF-κB kinase; Keap: 1 Kelch-like ECH-associated protein 1; NF-κBp65: Nuclear factor-kappa B; Nrf2: Nuclear factor erythroid 2-related factor 2; ROS: Reactive oxygen species; STZ: Streptozotocin; TNF-α: Tumor necrosis factor-alpha.

## Competing interests

The study was funded by Nutrition 21, Inc., NY, USA. Nutrition 21 also supplied the chromium picolinate and histidinate used in the study. James Komorowski is an employee of Nutrition 21, the distributors of chromium picolinate and histidinate under a license from the USDA.

## Authors' contributions

MYS, ZT and FA participated in data collection and laboratory analyses and assisted in every aspect of the study. BA and AD participated in study design and data interpretation and wrote the first draft of the manuscript. JRK and MA contributed to the study design, interpretation and preparation of the manuscript. KS participated in organization of the study and data interpretation and preparation of the manuscript. All authors read and approved the final manuscript.

## References

[B1] Barnard RJ Roberts CK Varon SM Berger JJ Diet-induced insulin resistance precedes other aspects of the metabolic syndrome J Appl Physiol 1998 84 1311 1315 9516198 10.1152/jappl.1998.84.4.1311

[B2] Buettner R Schölmerich J Bollheimer LC High-fat diet: modeling the metabolic disorders of human obesity in rodents Obesity 2007 15 798 808 10.1038/oby.2007.608 17426312

[B3] Broadhurst CL Domenico P Clinical studies on chromium picolinate supplementation in diabetes mellitus-a review Diabetes Technol Ther 2006 8 677 687 10.1089/dia.2006.8.677 17109600

[B4] Pop-Busui R Sima A Stevens M Diabetic neuropathy and oxidative stress Diabetes Metab Res Rev 2006 22 257 273 10.1002/dmrr.625 16506271

[B5] Ceriello A New insights on oxidative stress and diabetic complications may lead to a "causal" antioxidant therapy Diabetes Care 2003 26 1589 1596 10.2337/diacare.26.5.1589 12716823

[B6] Wiernsperger NF Oxidative stress as a therapeutic target in diabetes: revisiting the controversy Diabetes Metab 2003 29 579 585 10.1016/S1262-3636(07)70072-1 14707886

[B7] Warner DS Huaxin S Ines BH Oxidants, antioxidants and the ischemic brain J Exp Biol 2004 207 3221 3323 10.1242/jeb.01022 15299043

[B8] Fridlyand LE Philipson LH Oxidative reactive species in cell injury: mechanisms in diabetes mellitus and therapeutic approaches Ann NY Acad Sci 2005 1066 136 151 10.1196/annals.1363.019 16533924

[B9] Zhang L Zalewski A Liu Y Mazurek T Cowan S Martin JL Hofmann SM Vlassara H Shi Y Diabetes-induced oxidative stress and low-grade inflammation in porcine coronary arteries Circulation 2003 108 472 478 10.1161/01.CIR.0000080378.96063.23 12860917

[B10] Fernandez-Real JM Ricart W Insulin resistance and chronic cardiovascular inflammatory syndrome Endocr Rev 2003 24 278 301 10.1210/er.2002-0010 12788800

[B11] Esposito K Nappo F Marfella R Giugliano G Giugliano F Ciotola M Quagliaro L Ceriello A Giugliano D Inflammatory cytokine concentrations are acutely increased by hyperglycemia in humans: role of oxidative stress Circulation 2002 106 2067 2072 10.1161/01.CIR.0000034509.14906.AE 12379575

[B12] Evans JL Goldfine ID Maddux BA Grodsky GM Oxidative stres and stress-activated signaling pathways: a unifying hypothesis of type-2 diabetes Endocr Rev 2002 23 599 622 10.1210/er.2001-0039 12372842

[B13] Sriwijitkamol A Christ-Roberts C Berria R Eagan P Pratipanawatr T DeFronzo RA Mandarino LJ Musi N Reduced skeletal muscle inhibitor of kappaB beta content is associated with insulin resistance in subjects with type 2 diabetes: reversal by exercise training Diabetes 2006 55 760 767 10.2337/diabetes.55.03.06.db05-0677 16505240

[B14] Cai D Yuan M Frantz DF Melendez PA Hansen L Lee J Shoelson SE Local and systemic insulin resistance resulting from hepatic activation of IKK-beta and NF-kappaB Nat Med 2005 11 183 190 10.1038/nm1166 15685173 PMC1440292

[B15] Barnes PJ Karin M Nuclear factor-kappaB: a pivotal transcription factor in chronic inflammatory diseases N Engl J Med 1997 336 1066 1071 10.1056/NEJM199704103361506 9091804

[B16] Bi Y Meng-yin C Liang H Sun WP Chen X Zhu YH He XY Yu QQ Li M Weng JP Effect of early insulin therapy on nuclear factor-kappaB inflammatory pathway in liver of diabetic rat Zhonghua Nei Ke Za Zhi 2009 48 17 22 19484971

[B17] McCarty MF Nutraceutical resources for diabetes prevention--an update Med Hypotheses 2005 64 151 158 10.1016/j.mehy.2004.03.036 15533633

[B18] Shinde UA Goyal RK Effect of chromium picolinate on histopathological alterations in STZ and neonatal STZ diabetic rats J Cell Mol Med 2003 3 322 329 14594557 10.1111/j.1582-4934.2003.tb00233.x PMC6741326

[B19] Wang YQ Yao MH Effects of chromium picolinate on glucose uptake in insulin-resistant 3T3-L1 adipocytes involve activation of p38 MAPK J Nutr Biochem 2009 20 982 991 10.1016/j.jnutbio.2008.09.002 19195868

[B20] Kwon MJ Chung HS Yoon CS Ko JH Jun HJ Kim TK Lee SH Ko KS Rhee BD Kim MK Park JH The effect of chromium on rat insulinoma cells in high glucose conditions Life Sci 2010 87 401 404 10.1016/j.lfs.2010.08.001 20723550

[B21] Cefalu WT Wang ZQ Zhang XH Baldor LC Russell JC Oral chromium picolinate improves carbohydrate and lipid metabolism and enhances skeletal muscle Glut-4 translocation in obese, hyperinsulinemic (JCR-LA corpulent) rats J Nutr 2002 132 1107 1114 12042418 10.1093/jn/132.6.1107

[B22] Sahin K Onderci M Tuzcu M Ustundag B Cikim G Ozercan IH Sriramoju V Juturu V Komorowski JR Effect of chromium on carbohydrate and lipid metabolism in a rat model of type 2 diabetes mellitus: the fat-fed, streptozotocin-treated rat Metabolism 2007 56 1233 1240 10.1016/j.metabol.2007.04.021 17697867

[B23] Anderson RA Polansky MM Bryden NA Stability and absorption of chromium and absorption of chromium histidinate complexes by humans Biol Trace Elem Res 2004 101 211 218 10.1385/BTER:101:3:211 15564651

[B24] Vincent JB Elucidating a biological role for chromium at a molecular level Acc Chem Res 2000 33 503 510 10.1021/ar990073r 10913239

[B25] Cefalu WT Hu FB Role of chromium in human health and in diabetes Diabetes Care 2004 27 2741 2751 10.2337/diacare.27.11.2741 15505017

[B26] Wang H Kruszewski A Brautigan DL Cellular chromium enhances activation of insulin receptor kinase Biochemistry 2005 44 8167 8175 10.1021/bi0473152 15924436

[B27] Morris BW Kouta S Robinson R MacNeil S Heller S Chromium supplementation improves insulin resistance in patients with Type 2 diabetes mellitus Diabet Med 2000 17 684 685 11051290 10.1046/j.1464-5491.2000.00342.x

[B28] Yamamoto A Wada O Ono TJ Distribution and chromium-binding capacity of a low-molecular-weight, chromium-binding substance in mice Inorg Biochem 1984 22 91 102 10.1016/0162-0134(84)80018-5 6502162

[B29] Davis CM Vincent JB Chromium oligopeptide activates insulin receptor tyrosine kinase activity Biochemistry 1997 36 4382 4385 10.1021/bi963154t 9109644

[B30] Reed MJ Meszaros K Entes LJ Claypool MD Pinkett JG Gadbois TM Reaven GM A new rat model of type 2 diabetes: the fat-fed, streptozotocin-treated rat Metabolism 2000 49 1390 1394 10.1053/meta.2000.17721 11092499

[B31] Kume S Uzu T Araki S Sugimoto T Isshiki K Chin-Kanasaki M Sakaguchi M Kubota N Terauchi Y Kadowaki T Haneda M Kashiwagi A Koya D Role of altered renal lipid metabolism in the development of renal injury induced by a high-fat diet J Am Soc Nephrol 2007 18 2715 2723 10.1681/ASN.2007010089 17855643

[B32] Farombi EO Shrotriya S Na HK Curcumin attenuates dimethylnitrosamine-induced liver injury in rats through Nrf2-mediated induction of heme oxygenase-1 Food Chem Toxicol 2008 46 1279 1287 10.1016/j.fct.2007.09.095 18006204

[B33] Lowry OH Rosebrough NJ Farr AL Randall RJ Protein measurement with the folin phenol reagent J Biol Chem 1951 193 165 175 14907713

[B34] SAS SAS^® ^User's Guide: Statistics (Version 9th.) 2002 Statistical Analysis SystemInstitute Inc., Cary, NC, USA

[B35] Jain SK Kannan K Chromium chloride inhibits oxidative stress and TNF-α secretion caused by exposure to high glucose in cultured monocytes Biochem Biophys Res Commun 2001 289 687 691 10.1006/bbrc.2001.6026 11726202

[B36] Jain SK Lim G Chromium chloride inhibits TNF-α and IL-6 secretion in isolated human blood monoclear cells exposed to high glucose Horm Metab Res 2006 38 60 62 10.1055/s-2006-924981 16477544

[B37] Jain SK Rains JL Croad JL Effect of chromium niacinate and chromium picolinate supplementation on lipid peroxidation, TNF-a, IL-6, CRP, glycated hemoglobin, triglycerides and cholesterol levels in blood of streptozotocin-treated diabetic rats Free Radic Biol Med 2007 43 1124 1131 10.1016/j.freeradbiomed.2007.05.019 17854708 PMC3568689

[B38] Kuhad A Bishnoi Mahendra Tiwari Vinod Chopra Kanwaljit Suppression of NF-κβ signaling pathway by tocotrienol can prevent diabetes associated cognitive deficits Pharmacol Biochem Behav 2009 92 251 259 10.1016/j.pbb.2008.12.012 19138703

[B39] Preuss HG Grojec PL Lieberman S Anderson RA Effects of different chromium compounds on blood pressure and lipid peroxidation in spontaneously hypertensive rats Clin Nephrol 1997 47 325 330 9181280

[B40] Dogukan A Tuzcu M Juturu V Cikim G Ozercan I Komorowski J Sahin K Effects of chromium histidinate on renal function, oxidative stress, and heat-shock proteins in fat-fed and streptozotocin-treated rats J Ren Nutr 2010 20 112 120 10.1053/j.jrn.2009.04.009 19616452

[B41] Figueroa-Romero C Sadidi M Feldman EL Mechanisms of disease: The oxidative stress theory of diabetic neuropathy Rev Endocr Metab Disord 2008 9 301 314 10.1007/s11154-008-9104-2 18709457 PMC4239697

[B42] Chen L Zhang J Zhang Y Wang Y Wang B Improvement of inflammatory responses associated with NF-kB pathway in kidneys from diabetic rats Inflamm Res 2008 57 199 204 10.1007/s00011-006-6190-z 18465086

[B43] Ma Q Battelli L Hubbs AF Multiorgan autoimmune inflammation, enhanced lymphoproliferation, and impaired homeostasis of reactive oxygen species in mice lacking the antioxidant-activated transcription factor Nrf2 Am J Pathol 2006 168 1960 1974 10.2353/ajpath.2006.051113 16723711 PMC1606627

[B44] He X Chen MG Ma Q Activation of Nrf2 in defense against cadmium-induced oxidative stress Chem Res Toxicol 2008 21 1375 1383 10.1021/tx800019a 18512965

[B45] Palsamy P Subramanian S Resveratrol protects diabetic kidney by attenuating hyperglycemia-mediated oxidative stress and renal inflammatory cytokines via Nrf2-Keap1 signaling Biochim Biophys Acta 2011 1812 719 731 21439372 10.1016/j.bbadis.2011.03.008

[B46] Tuzcu M Sahin N Orhan C Agca CA Akdemir F Tuzcu Z Komorowski J Sahin K Impact of chromium histidinate on high fat diet induced obesity in rats Nutr Metab (Lond) 8 8 28 10.1186/1743-7075-8-28 PMC3094204 21539728

[B47] Akude E Zherebitskaya Elena Subir K Chowdhury Roy Girling Kimberly Fernyhough Paul 4-Hydroxy-2-Nonenal Induces Mitochondrial Dysfunction and Aberrant Axonal Outgrowth in Adult Sensory Neurons that Mimics Features of Diabetic Neuropathy Neurotox Res 2010 17 28 38 10.1007/s12640-009-9074-5 19557324 PMC2894940

[B48] Praticò D Tangirala RK Rader DJ Rokach J FitzGerald GA Vitamin E suppresses isoprostane generation in vivo and reduces atherosclerosis in Apo E-deficient mice Nat Med 1998 4 1189 1192 10.1038/2685 9771755

